# Does Creativity Influence Visual Perception? - An Event-Related Potential Study With Younger and Older Adults

**DOI:** 10.3389/fpsyg.2021.742116

**Published:** 2021-10-18

**Authors:** Petra Csizmadia, István Czigler, Boglárka Nagy, Zsófia Anna Gaál

**Affiliations:** ^1^Institute of Cognitive Neuroscience and Psychology, Research Centre for Natural Sciences, Budapest, Hungary; ^2^Doctoral School of Psychology (Cognitive Science), Budapest University of Technology and Economics, Budapest, Hungary

**Keywords:** creativity, aging, perception, ambiguity, ERP, decoding

## Abstract

We do not know enough about the cognitive background of creativity despite its significance. Using an active oddball paradigm with unambiguous and ambiguous portrait paintings as the standard stimuli, our aim was to examine whether: creativity in the figural domain influences the perception of visual stimuli; any stages of visual processing; or if healthy aging has an effect on these processes. We investigated event related potentials (ERPs) and applied ERP decoding analyses in four groups: younger less creative; younger creative; older less creative; and older creative adults. The early visual processing did not differ between creativity groups. In the later ERP stages the amplitude for the creative compared with the less creative groups was larger between 300 and 500 ms. The stimuli types were clearly distinguishable: within the 300–500 ms range the amplitude was larger for ambiguous rather than unambiguous paintings, but this difference in the traditional ERP analysis was only observable in the younger, not elderly groups, who also had this difference when using decoding analysis. Our results could not prove that visual creativity influences the early stage of perception, but showed creativity had an effect on stimulus processing in the 300–500 ms range, in indexing differences in top-down control, and having more flexible cognitive control in the younger creative group.

## Introduction

Creativity, the ability to create novel, original, appropriate and also useful ideas or products (Stein, 1953; Runco and Jaeger, 2012), is one of the most important life skills. In facilitating adaptive behavior to a changing environment it had a significant role, not only in evolution, but also in the development of human civilization. In addition, creativity has an essential role in solving everyday problems and tasks. Despite its popularity, we do not know enough about its cognitive background and the neural mechanisms underlying creativity, which are partly the result of issues and limitations from the electrophysiological methods used. These limitations are: strict and unusual laboratory environment; the forbidden movement and speech to eliminate artifacts; the time-bound paradigms that require a large number of repetitions; and the time pressure (1–3 min) to complete a task. These are all disadvantages which hinder the study of the creative process in cognition, and help to explain why there are so few studies to date examining creative processes using electroencephalography. In the current study, we chose not to exploit directly the neurobiological background of creativity due to these limitations, but rather to focus on whether creativity begins with the perception of stimuli, or as to whether this plays a part in creativity as a cognitive process. Consequently, we chose to compare creative and less creative people to see if they already differ in their early processes, or only later top-down processes cause a difference in their perception, or the perception of stimuli is not different at all.

Since creativity is a complex construct and hard to quantify, most studies have chosen only to examine divergent and convergent thinking – two measurable cognitive processes having a central role in creative cognition (Guilford, 1967). Divergent thinking is a crucial part of open-ended problem solving as it requires mental flexibility to come up with as many ideas as possible to generate novel ideas. By contrast, convergent thinking is a crucial part of closed-ended problem solving being the ability to: reason logically; to recognize rules; and to evaluate and choose the most appropriate solution from different ideas. So, convergent as opposed to divergent thinking relies more on persistence, mental focus and cognitive control. These two ways of thinking are both important, but occur in different stages of the creative process with varying degrees of significance. This has been described as dynamic changes in combination with balance between flexibility and persistence (Dual Pathway of Creativity; Nijstad et al., 2010; Metacontrol State Model; Hommel, 2015; neurocognitive framework of the metacontrol of creative cognition; Zhang et al., 2020), or as flexible cognitive control (Zabelina and Robinson, 2010).

The process of creative problem solving consists of several stages: it starts with the discovery of the problem; followed by the generation of ideas; incubation; evaluation of the ideas; selection of the appropriate solution; and then, elaboration of the selected outcome (Wallas, 1926). Since the first step in creative problem solving is to detect the problem, it is particularly important to determine how a person perceives, or senses a given problem, as this governs the way they think, and this will affect all the subsequent stages in the creative process (Nęcka, 2011). Seeing and interpreting stimuli in different individual ways than others (perceptual restructuring) is one element for generating creative ideas (Wiseman et al., 2011). At this stage, it is advantageous if the attention is not too focused in order for more information to be processed. Also, a broader perception of the environment is considered essential in creative cognition.

Based on the inhibition deficit theory of aging – which hypothesize an age-related decline in inhibitory processes (Hasher and Zacks, 1988) – and the load theory of attention (Lavie, 2005) – which claims insufficient cognitive control leads to greater distractor processing – we can assume that older adults are not able to disregard task-irrelevant environmental stimuli effectively. Thus, the question arises as to whether the decline in inhibitory processes associated with aging may lead to a widened attentional focus and hence changes in perception which might offer some benefit in the initial stage^1^ of creative problem solving for the elderly. To study this question, we compared both less and more creative younger and older adults in four groups.

Several studies have examined the potential benefits of distractibility. For example, Kim et al. (2007) and Healey et al. (2008) found that if previously task-irrelevant information becomes task-relevant, the elderly show a priming effect and with this they gain a benefit compared to younger adults, who do not. The results of Radel et al. (2015) suggest that disinhibition can be an advantage by facilitating idea generation in the early phase of the creative process, although only younger adults participated in their study. Taken together, this as well as The Matched Filter Hypothesis from Chrysikou et al. (2014) – which claims the optimal level of cognitive control depends on a given task and goal – let us to hypothesize that a decreased cognitive control in older adults would facilitate their visual processing, especially in the creative older group. Still, we should note an important difference between younger and older adults’ broader perception of the environment; which in the former, it may be due to an increase in flexibility; while in the latter, this may depend more on a decrease in mental flexibility. Consequently, if we study the other stages of the creative process then the expected benefit in the early stage can turn into a disadvantage later in the older group.

Because of the difficulty in directly measuring creative processes with EEG, several studies have chosen to consider divergent thinking and the capability to create original products as an indicator of creativity and to correlate test scores with cognitive functions as measured by their research paradigm. For example, Zabelina and Ganis (2018) in using an oddball paradigm found a positive correlation between divergent thinking and the N2 component, thus supporting a significant role for cognitive control in the process of creativity. In our previous study (Nagy et al., under review^2^) we found decreased and less flexible cognitive control for creative older adults when compared with less creative elderly and young participants in a task-switching paradigm. Our conclusion was that less effective inhibitory control enhances creativity in older adults. One of the few studies to directly examine an important process of creative cognition is by Rutter et al. (2012) in which they established a relationship between conceptual expansion – which can arise in both divergent and convergent thinking – and the N400 component. Specifically, they found the measurable effort needed to form a connection between two unrelated concepts in a greater N400 amplitude for creative (novel metaphorical) compared to literal (everyday) phrases.

Despite the most commonly used method for studying creativity from an electrophysiological standpoint being the analysis of oscillatory EEG activity (time-frequency decomposition, spectral power density – for reviews see Dietrich and Kanso, 2010; Stevens and Zabelina, 2019); we opted for the less usual method using the event-related potential (ERP) technique to provide direct information about specific stages of cognitive processing as a function of participants’ creativity, and the effect of creativity on perception. We initially studied the P1 and N1 components for 200 ms, but after 200 ms, we examined the long-lasting positivity in 100 ms time windows in the 200-600 ms range. The visual P1 and N1 are exogenous sensory components and they appear at about 100 ms after stimulus presentation with positive and negative polarity, respectively (Luck, 2014). These components are correlates of the primary visual response and sensory processing (Mangun and Hillyard, 1990), although top-down processes such as attention (Paz-Caballero and García-Austt, 1992; Hillyard et al., 1998; Luck et al., 2000); and arousal (Vogel and Luck, 2000) may also modulate their parameters. The maximum amplitude of the P1 component can usually be derived from the lateral occipital electrodes 100–130 ms after stimulus onset, and due to its extrastriatal origin, P1 is sensitive to the physical parameters (e.g., contrast) of the stimuli. There are several visual N1 subcomponents, and they can be measured at anterior or parietal or lateral occipital scalp areas (Luck, 2014). For us the lateral occipital N1 component was considered to be relevant as it could be influenced by both the physical properties of the stimulus and spatial attention, and it appears to be involved in early discrimination processes (Vogel and Luck, 2000; Hopf et al., 2002). Time windows after 200 ms already indicate cognitive processes related to later stages of visual processing, such as: categorization, working memory, or decision processes. These later components are also called as endogenous cognitive components, which reflect task-related neural processes (Luck, 2014). In assuming that creativity has an effect on perception, we expected to find differences between the less creative and creative groups already in the parameters of the early components, which we hypothesized could influence the later stages of stimulus processing as well, from the observation of amplitude differences.

From younger and older adults we separated two groups, creative and less-creative, by using two figural subtests of the Hungarian analog of the Torrance Test of Creative Thinking (Fáy et al., in press). We considered creativity to be domain specific (Baer, 2015); and as only visual stimuli were presented in our active oddball paradigm, we used only the figural subtests of the TTCT. The ratio of standard and deviant stimuli was 90% and 10%, and deviant stimuli (target stimuli) were images of butterflies, where the subjects’ task was to press a button when they saw one (for details of oddball paradigms see Polich, 2007). The real purpose of our study was to focus on the two types of standard stimuli by presenting 50-50% unambiguous and ambiguous portrait paintings, for which no response action was required. Our design followed ERP studies on face perception by Bentin et al. (1996), in which the to-be analyzed stimuli appeared frequently, but did not require an overt or covert response (see also Czigler et al., 2007).

In perception studies, ambiguous images are often used as they can be perceived in multiple ways, the perceptual interpretation changes periodically although the visual content does not change (Kornmeier and Bach, 2012). This characteristic is suitable for revealing how sensory and cognitive processes interplay during perception (Long and Toppino, 2004). The main theories consider the possibility either of bottom-up or top-down underlying processes, or a combination of the two. The bottom-up (sensory) approach assumes that perceptual switches are the result of the alternation of passive adaptation, recovery, and mutual inhibition of competing neural elements in early visual areas; while the top-down (cognitive) approach is supported by findings that reveal the role of volition, attention and experience in reversals (for reviews see Long and Toppino, 2004; Kornmeier and Bach, 2012). With a focus on our experiment, we have to be aware of the critical role of inhibition in bistable perception: only one percept is visible at a time, during which its competitor has to be suppressed, and this can happen in several stages according to the multistage hybrid models (Alais, 2012). The assumed age-related decline of inhibitory processes (Hasher and Zacks, 1988) made us hypothesize that older adults would perceive more details when presented with ambiguous paintings.

After performing traditional ERP (univariate) analysis to test our hypotheses, we then used ERP-decoding (Bae and Luck, 2018), which is a multivariate pattern analysis method. The term “multivariate pattern analysis” (MVPA) includes different methods for analyzing neuroimaging data. Their main common feature is that they consider the relationships between multiple variables (e.g., channels) at the same time, instead of treating them as independent by assessing the relative activation magnitude. Decoding is one of the most common applications of MVPA, the term “decoding” refers to the prediction of experimental conditions (e.g., trial types or stimulus types) from patterns in neural data (Grootswagers et al., 2017). Either MVPA or decoding methods are commonly used to examine fMRI data (for reviews see e.g., Norman et al., 2006; Tong and Pratte, 2012; Haynes, 2015). While decoding methods for time series data as MEG and EEG (for review see: Grootswagers et al., 2017) have only recently been applied to answer experimental questions in cognitive neuroscience, they have been quite commonly used over the past years in brain-computer interfaces (for reviews see e.g., Curran and Stokes, 2003; Müller et al., 2008; Chamola et al., 2020).

The main differences between univariate and multivariate methods are in their sensitivity and approach to experimental questions. Multivariate methods may show differences that would not be apparent in univariate analysis because the activity pattern across electrodes can be better at separating two conditions than the voltages measured at individual locations. Multivariate decoding can also use information that would not be detectable when comparing, for example, ERPs in a univariate analysis (Grootswagers et al., 2017; for details see Hebart and Baker, 2018). Regarding the conceptual background behind the experimental questions, the univariate method is an activation-based, while the multivariate analysis is an information-based approach. In the former (activation-based) case, we are interested in whether there is a difference between the amplitude and latency of the components belonging to different conditions, so we need to analyze the stages of the given cognitive process. In the latter (information-based) case, we need to ask whether or not the neural signal contains information about the condition or trial type (Hebart and Baker, 2018).

In summary, our main goal was to reveal whether visual creativity begins with the perception of stimuli, or whether it plays no part in creativity as a cognitive process; as well as, to study whether healthy aging has an effect on these processes. To answer these questions we examined event related potentials evoked by unambiguous and ambiguous portrait paintings in four groups being younger less creative, younger creative, older less creative and older creative. According to the hypothesis that disinhibition can be an advantage by facilitating idea generation in the early phase of the creative process, we assumed that decreased cognitive control in older adults would facilitate their visual processing, especially in the creative older group; and since inhibition plays a critical role in bistable perception, we hypothesized that the elderly would perceive more details from ambiguous images.

Furthermore, we used ERP decoding in an exploratory manner, with the aim of determining whether the signal contained information about the ambiguity of the stimulus; and if so, whether creativity or age influenced stimulus processing and representation. If we can decode (indexed by decoding accuracy significantly higher than chance) the stimulus category from the neural signal, we can be more confident that the signal really represents the stimulus, and not some other irrelevant processes.

## Materials and Methods

### Participants

In our study there were 36 younger (aged 18–30 years) and 38 older adults (aged 60–75 years) participating, and because of technical issues the results of two younger and three older participants were excluded. Age groups were separated into three groups according to their Creativity Index (CI) scores with the less creative on the one hand and most on the other, and with those in the middle group, between the less and the more creative groups, being discarded from the study – resulting in there being 12 participants in each of the four groups. To measure their creativity, we used the updated and standardized version of the Figural Subtest of Barkóczi-Klein Creativity Test (Hungarian analog of the Torrance Test of Creative Thinking; Barkóczi and Klein, 1968; Barkóczi and Zétényi, 1981) by Fáy et al. (in press) (this manual is available on request). To rule out dementia-related differences between younger and older adults, intelligence was measured by four subtests representing the four major components (Similarities – verbal comprehension, Digit Span – working memory, Matrix Reasoning – perceptual reasoning, Coding – processing speed) of the Hungarian version of WAIS-IV (Rózsa et al., 2010). Both age groups had higher scores on the subtests than the average for the population. The demographic data of the four groups, IQ test and CI scores are summarized in Table 1.

**TABLE 1:** Demographic data and test scores for the four creativity groups based on age (mean and standard deviation).

	Number of participants	Age (years)	Education (years)	WAIS-IV Subtests				Creativity Index
				S	DS	MR	C	
Younger less-creative group	12 (9 female)	21.83 ± 1.27	14.92 ± 1.24	11.08 ± 2.71	10.08 ± 2.19	11.58 ± 2.57	12.92 ± 2.81	0.77 ± 0.17
Younger more-creative group	12 (7 female)	23.08 ± 2.81	16.08 ± 2.15	12.33 ± 2.31	11.50 ± 3.15	11.83 ± 3.16	11.83 ± 2.89	1.41 ± 0.17
Older less-creative group	12 (6 female)	69.25 ± 2.90	16.83 ± 3.49	12.83 ± 1.95	10.83 ± 2.29	11.25 ± 2.90	13.75 ± 2.38	1.34 ± 0.15
Older more-creative group	12 (7 female)	67.25 ± 4.11	16.25 ± 3.14	12.92 ± 2.71	13.33 ± 3.94	13.42 ± 2.47	15.83 ± 2.69	1.95 ± 0.19

*S, Similarities; D, Digit Span; MR, Matrix Reasoning; C, Coding.*

All participants were right-handed, had normal or corrected-to-normal vision (at least 5/5 in a version of the Snellen charts), and were free of any kind of neurological or psychiatric disorder. All participants were paid for their contribution, and written and informed consent was obtained from all participants prior to their participation. The study was approved by the Joint Psychological Research Ethics Committee (EPKEB, Hungary).

### Creativity Index

We calculated the Creativity Index based on a method from Fáy and Jeney (2016). The variables were fluency (F), originality (O), elaboration (E), resistance to premature closure (C) and creative strengths (CS). In order to reduce the high correlation between fluency and other variables, they were all divided by the fluency score. The participants’ variable scores were normalized with the representative age group’s average, since we did not examine how creative variables change with age, but whether there are differences within age groups in the visual and cognitive processes between less creative and creative individuals, and whether and how this difference alters between young and elderly people. We calculated the Creativity Index as (CR1 + CR2 + CS)/3, where CR1 was the score from the Circles task (CR1 = (F + O/F + E/F)/3); and CR2 was the score from the Incomplete figures task (CR2 = [(E/F + C/F)/2 + F + O/F)/3]; and thirdly, CS was the creative strengths score.

Here we should emphasize that as we normalized the scores with the representative age group’s average, there are different raw scores behind similar scaled scores in the younger and older groups.

### Stimuli and Procedure

**FIGURE 1:**
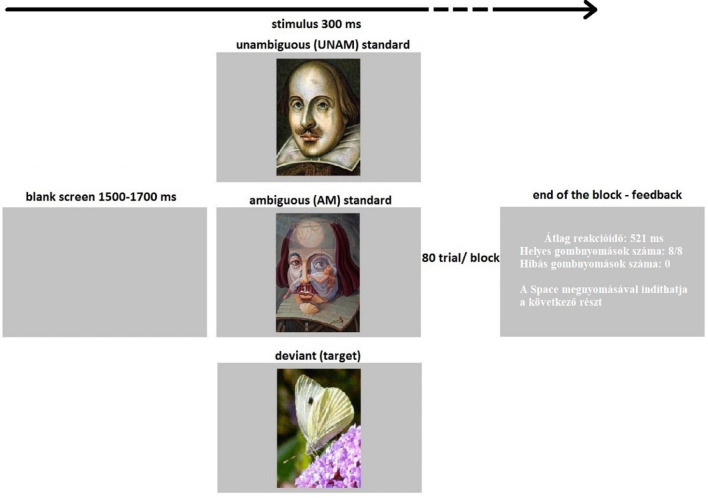
Illustration of the sequence and timing of stimuli: The target (10%, a butterfly) which the participants had to press a button for, or a standard stimulus (45–45% unambiguous or ambiguous portraits) which no reaction was needed, was presented for 300 ms. An inter-stimulus interval with a blank screen lasted for 1500–1700 ms. Participants got feedback at the end of the block of 80 trials. The feedback contained average reaction time, number of correct and incorrect button presses and an instruction to “Press Space to start the next section”. The experiment contained six blocks.

The experimental stimuli were presented with MATLAB R2016b (The MathWorks, Inc.) on a 21.5-in. LCD monitor (Asus VS229na, 60-Hz refresh rate), on a gray (44.48 cd/m^2^) background, at a viewing distance of 1.44 m. The experimental design is shown in Figure 1. Participants had to perform an active visual oddball task. The stimuli were colored pictures of: eight butterflies, as deviants; and as standards four unambiguous along with four ambiguous portrait paintings^3^. The size of the images were 200 × 280 pixels (2,13° × 3,99°), and the stimuli appeared on the center of the screen. The participant’s task was to press the Space button (with both hands) as soon as a butterfly appeared on the screen. There were 80 trials (8 deviants and 72 standards) within a block. A deviant butterfly was not repeated, a standard portrait was repeated 9 times within a block. The two types of standard stimuli (unambiguous and ambiguous) were equally presented. In total, there were 48 deviant, 216 unambiguous standard and 216 ambiguous standard trials from six blocks. Stimulus duration was 300 ms, and the inter-stimulus intervals were between 1,500 and 1,700 ms with a jitter in steps of 50 ms. The presentation order of the stimuli was pseudorandom with the restriction that a minimum of 4, and a maximum of 12, subsequent standard stimuli would appear between two deviants; and a maximum of 3 standard stimuli, of the same type, would follow each other. The feedback given after each block was: the average reaction time; the number of correct responses and errors. The experiment started with a practice block (20 trials), in which EEG was not recorded. In the practice sequence butterfly and portrait pictures were not the same as those used during the EEG recording, and only unambiguous and not ambiguous standards were presented. The purpose of the practice session was only that the participants understand the task, which was responding to the butterfly, so the type of standard stimulus was irrelevant in this respect.

### EEG Recording

Electrophysiological recording was performed in an electrically and acoustically shielded room. Electrical brain activity was recorded from 32 locations according to the extended 10-20 system (BrainVision Recorder 1.21.0303, ActiChamp amplifier, Ag/AgCl active electrodes, EasyCap (Brain Products GmbH), sampling rate: 1000 Hz, DC-70 Hz online filtering). The ground electrode was placed on the forehead (AFz) and the reference electrode was on the nose tip. Both horizontal and vertical electrooculogram (HEOG and VEOG) were recorded with bipolar configurations between two electrodes (placed lateral to the outer canthi of the two eyes and above and below the left eye, respectively).

### Data Analysis

The EEG signal was bandpass filtered offline with a non-causal Kaiser-windowed Finite Impulse Response filter (low pass filter parameters: 30 Hz of cutoff frequency, beta of 12.2653, a transition bandwidth of 10 Hz; high pass filter parameters: 0.1 Hz of cutoff frequency, beta of 5.6533, a transition bandwidth of 0.2 Hz) in MATLAB environment (The MathWorks, Inc.). In order to reject eye-movement artifacts (blinking, looking aside) Independent Component Analysis (ICA) was applied on the filtered EEG data, which was performed with the EEGLAB toolbox (Delorme and Makeig, 2004).

Epochs ranging from −100 to 1,000 ms relative to the onset of stimuli were extracted for all deviants and standards. The first 100 ms of each epoch served as the baseline. Epochs with larger than 100 μV, or smaller than 2 μV voltage change, were considered artifacts and rejected from any further processing.

In this paper we discuss only the results from unambiguous and ambiguous standard trials.

#### ERP Data Analysis

Three regions of interest (ROI-s) were calculated based on scalp topographies: the left parieto-occipital ROI comprising the P7, P3, PO7, PO3, and O1 locations; the middle parieto-occipital ROI comprising the Pz, POz, and Oz locations; and the right parieto-occipital ROI comprising the P8, P4, PO8, PO4 and O2 electrodes.

P1 latency was measured as the largest positivity within the 30-130 ms range, and P1 amplitude was measured as the mean amplitude within ± 5 ms around peak latency for each participant. It is likely that a positivity superimposed on the negativity following the P1 component and since the extent of this differed between the younger and older groups, we did not measure the peak latency and amplitude of the N1 component, but P1-N1 peak to peak, and N1- 200-300 ms positivity peak to peak amplitudes for each participant, where N1 was defined as the largest negativity within the 100–200 ms range. Between 200–600 ms interval we measured the mean amplitudes within 200–300, 300–400, 400–500 and 500–600 ms ranges. The latencies and amplitudes were measured at left-, middle- and right parieto-occipital ROI-s.

Statistical analyses were performed with Statistica 13 (TIBCO Software Inc.). Mixed ANOVAs for ERPs were performed with *Age* (younger/older) and *Creativity* (less creative/creative) as between-subject factors; and *Stimulus* (unambiguous/ambiguous) and *ROI* (left-/middle-/right parieto-occipital) as within-subject factors.

*Post hoc* analysis was performed by using the Tukey honest significant difference (HSD) test, and the effect size was calculated as partial eta square (η_p_^2^).

#### ERP Decoding

We decoded the ambiguity of the standard stimuli on the basis of the scalp distribution of the ERP signal. The method of Bae and Luck (2018) and their Matlab scripts^4^ were used. By down-sampling the data and averaging trials (belonging to the same stimulus type) we were able to improve the signal-to-noise ratio (Grootswagers et al., 2017; Bae and Luck, 2018); therefore, the decoding was performed on averages rather than on single-trial data, and only on every fourth time point (1 data point per 4 ms – 250 Hz) of the data. Decoding was conducted separately for each participant, and independently at each of the 275 time points from −100 to + +1000 ms. Twenty-seven electrodes (F7, F3, FC3, C3, FZ, CZ, F8, F4, FC4, C4, CP5, CP6, P7, P3, PZ, P4, P8, PO7, PO3, POZ, PO4, PO8, O1, OZ, O2, T7, T8) were used for the analysis, and HEOG-s and VEOG-s were excluded.

As in Bae and Luck previous decoding studies (Bae and Luck, 2018, 2019b, c), the combination of support vector machine (SVM) and error-correcting output codes (ECOC, Dietterich and Bakiri, 1995) were used to classify the ambiguity of the standard stimuli on the basis of the spatial distribution of the signal over the 27 scalp electrodes. The epoched data was organized based on the ambiguity, and then the trials were randomly divided into three equal-sized sets of approximately 70 trials (3 groups of approx. 70 trials for both stimuli types). Of note, this number varied slightly from subject to subject due to the different number of trials thrown out during artifact rejection. The trials for a given stimulus type in each group/set were averaged together, creating a scalp distribution for the time point being analyzed (a matrix of 3 sets × 2 stimuli type × 27 electrodes). The averaged data from two sets of the three groups belonging to the given stimulus type were used as a training dataset, and the third was used to test the classifier performance. Decoding accuracy was computed by comparing the true stimulus type with the predicted type. Since the classification was binary and both possibilities were equally probable, chance performance was 0.5. This procedure was repeated three times (3-fold-cross-validation), with each of the three sets of data for both stimulus type serving as the testing dataset. To provide a more robust estimation of decoding accuracy, this entire procedure was iterated 50 times using new random assignments of trials for the three data sets. After finishing all iterations of the cross-validation procedure, we got a decoding percentage for a given time point based on 300 decoding attempts from collapsing decoding accuracy across the two stimulus types, and across the three cross-validations, as well as across the 50 iterations (2 stimulus type × 3 cross validations × 50 iterations). As mentioned above, the decoding procedure was applied separately at each time point for a given participant, producing one decoding accuracy value for each time point (aggregated across cross-validations and iterations). Finally, to minimize noise, the decoding accuracy values were smoothed across time points using a 5-point moving window (equivalent to a time window of ± 8 ms).

For statistical analysis of decoding accuracy we used a non-parametric cluster-based permutation analysis, which is at the same time an appropriate procedure to controlling for multiple comparisons (Bae and Luck, 2019a, b). To do this, we also used Bae and Luck’s Matlab scripts. This method includes three steps. First, one-sample t-tests were used to determine whether decoding accuracy at each individual time point was significantly greater than chance (0.5). Second, for the found clusters of contiguous time points for which the single point t tests were significant (*p* < 0.05) cluster-level t mass (the sum of the t scores within each cluster) was computed. Thirdly, a given cluster mass was compared to the mass that would be expected by chance, as determined via permutation tests. Permutation tests were used to establish a null distribution of cluster-level t mass values to determine whether a given cluster mass was larger than expected by chance. Permutation was conducted at the testing stage of the decoder output rather than at the training stage of the decoder (for detailed description see Bae and Luck, 2019b). The statistical analysis was performed separately for the four groups.

To compare our groups’ decoding accuracy, we aggregated decoding accuracy across time points, specifically during five periods (100–200, 200–300, 300–400, 400–500, 500-600 ms) within those groups, which mostly had statistically significant results (greater than chance decoding accuracy). To aggregate across time points during these periods, we averaged the decoding accuracy across the given time points which was done separately for each participant. Then repeated measures of ANOVAs for these values were performed with *Age* (younger/older) and *Creativity* (less creative/creative) as between-subject factors, and *Time* (five periods) as within-subject factor. *Post hoc* analysis was performed by using the Tukey honest significant difference (HSD) test.

## Results

### Behavioral Data

With the exception of a few mistakes, omitting a button press or an extra button press (on average less than 1.3% of the trials), participants completed the tasks correctly, and our behavioral results showed that participants performed their tasks properly and attentively.

The average reaction time and standard deviation was 475.72 ± 28.60 ms in the younger less creative group, 422.31 ± 27.49 ms in the younger more creative group, 500.36 ± 48.71 ms in the older less creative group and 484.39 ± 27.28 ms in the older more creative group. Analyzing the average reaction times of the 6 blocks by factorial ANOVA, we found that younger adults responded faster than older adults when the targets appeared (*Age* main effect: *F*(1,44) = 19.24, *p* < 0.001, η_p_^2^ = 0.30). We also found that the less-creative groups were slower than the more creative groups (*Creativity* main effect: *F*(1,44) = 12.31, *p* = 0.001, η_p_^2^ = 0.22); but this effect was significant only in the younger group (*p* = 0.002) according to the *post hoc* test of the *Age x Creativity* interaction (*F*(1,44) = 3.59, *p* = 0.065, η_p_^2^ = 0.08), not in the older group (*p* = 0.666).

### Event-Related Potentials

The mean amplitudes of the investigated components and time windows in the four groups measured at three parieto-occipital ROIs and the three fronto-central ROIs for the N1 component in the younger groups as shown in Table 2.

**TABLE 2:** Mean amplitudes and standard deviations of the P1 component, P1-N1 and N1- 200–300 ms positivity peak to peak and the 100 ms-long time windows elicited by unambiguous (UNAM) and ambiguous (AM) stimuli in the four groups.

		P1			P1-N1 peak to peak			N1- 200–300 ms positivity peak to peak			200–300 ms			300–400 ms			400–500 ms			500–600 ms		
							
		Left ROI	Mid. ROI	Right ROI	Left ROI	Mid. ROI	Right ROI	Left ROI	Mid. ROI	Right ROI	Left ROI	Mid. ROI	Right ROI	Left ROI	Mid. ROI	Right ROI	Left ROI	Mid. ROI	Right ROI	Left ROI	Mid. ROI	Right ROI
Younger less creative group	UNAM	4.10 ± 1.69	3.77 ± 2.55	6.68 ± 3.94	−6.24 ± 3.56	−5.14 ± 1.75	−7.35 ± 4.45	11.40 ± 4.66	9.10 ± 3.02	12.31 ± 3.98	9.26 ± 5.27	7.72 ± 4.32	11.64 ± 5.95	6.68 ± 4.22	6.01 ± 4.48	9.15 ± 5.11	4.52 ± 3.09	4.22 ± 3.65	5.12 ± 3.66	2.90 ± 2.12	3.19 ± 2.66	2.77 ± 2.77
	AM	4.02 ± 2.03	3.58 ± 2.85	6.73 ± 3.98	−6.15 ± 3.56	−5.24 ± 1.58	−6.92 ± 4.35	11.63 ± 5.25	9.31 ± 3.77	12.06 ± 3.98	9.50 ± 5.39	7.65 ± 4.45	11.87 ± 5.97	7.61 ± 5.05	6.19 ± 5.25	10.06 ± 6.11	5.27 ± 3.54	4.28 ± 3.98	5.88 ± 4.20	3.66 ± 2.25	3.19 ± 2.62	3.59 ± 3.00
Younger more creative group	UNAM	6.27 ± 3.13	4.49 ± 2.15	7.40 ± 2.87	−4.85 ± 2.93	−4.86 ± 2.87	−7.25 ± 2.90	8.56 ± 3.47	8.30 ± 4.22	9.99 ± 3.84	11.96 ± 3.98	7.93 ± 4.03	12.11 ± 4.46	9.59 ± 4.06	8.65 ± 3.04	11.84 ± 4.35	6.00 ± 2.22	5.95 ± 2.30	7.25 ± 2.13	3.02 ± 1.48	3.84 ± 2.13	3.92 ± 1.88
	AM	6.38 ± 3.35	4.03 ± 2.62	7.69 ± 3.18	−4.70 ± 2.07	−4.50 ± 2.65	−7.52 ± 2.83	8.20 ± 2.98	8.27 ± 4.37	11.30 ± 3.38	9.89 ± 3.83	7.79 ± 3.95	11.47 ± 4.41	10.77 ± 4.17	9.02 ± 3.05	12.36 ± 4.25	7.54 ± 2.20	6.67 ± 1.84	8.19 ± 2.40	4.68 ± 1.56	4.73 ± 1.68	5.15 ± 1.94
Older less creative group	UNAM	6.36 ± 2.95	3.44 ± 2.89	6.85 ± 3.42	−13.14 ± 6.18	−6.15 ± 3.97	−12.92 ± 5.70	12.50 ± 4.88	6.98 ± 3.76	11.76 ± 4.50	5.71 ± 2.95	4.28 ± 2.03	5.70 ± 2.76	4.22 ± 2.87	4.55 ± 2.59	4.74 ± 3.11	4.15 ± 2.95	5.66 ± 2.84	4.12 ± 3.13	2.64 ± 3.28	4.30 ± 3.26	2.74 ± 3.42
	AM	6.31 ± 3.00	3.32 ± 2.99	6.68 ± 3.37	−13.07 ± 5.99	−6.49 ± 4.15	−12.76 ± 5.98	11.95 ± 4.79	7.16 ± 3.68	11.02 ± 4.47	5.18 ± 2.79	3.98 ± 2.04	4.94 ± 2.65	4.05 ± 2.57	4.59 ± 2.25	4.59 ± 2.91	4.35 ± 2.70	5.97 ± 2.69	4.46 ± 3.03	3.14 ± 3.38	4.88 ± 3.35	3.38 ± 3.35
Older more creative group	UNAM	4.30 ± 2.31	3.24 ± 2.43	4.79 ± 2.49	−8.62 ± 4.56	−5.27 ± 1.78	−10.36 ± 6.31	11.09 ± 5.34	7.71 ± 4.37	12.09 ± 6.84	6.76 ± 2.92	5.68 ± 3.39	6.52 ± 3.76	5.89 ± 4.06	6.17 ± 4.22	5.81 ± 4.29	4.86 ± 3.22	6.26 ± 3.29	4.51 ± 3.05	3.61 ± 2.39	5.13 ± 2.45	3.52 ± 2.16
	AM	4.26 ± 2.51	3.10 ± 2.55	4.79 ± 2.06	−9.02 ± 4.87	−5.59 ± 2.21	−10.49 ± 6.15	11.44 ± 5.72	8.18 ± 5.12	11.82 ± 6.70	6.67 ± 2.87	5.69 ± 3.16	6.12 ± 3.66	6.01 ± 3.86	6.28 ± 3.89	5.90 ± 4.12	5.70 ± 3.16	6.97 ± 3.20	5.05 ± 3.28	4.50 ± 2.08	5.93 ± 2.27	4.22 ± 2.05

*The data were measured at parieto-occipital ROIs.*

**FIGURE 2:**
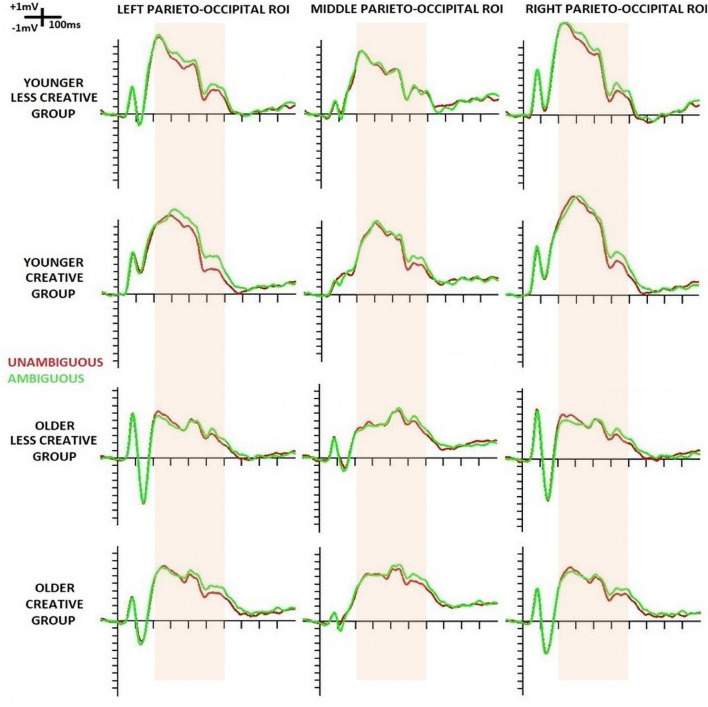
Event-related potentials (ERPs) at left-, middle- and right parieto-occipital ROIs evoked by the standard stimuli in the four groups (younger – less creative, younger – creative, older – less creative, older – creative). ERPs for unambiguous portraits are displayed in red and for ambiguous portraits in green. The pink band shows the period in which ERP was analyzed in 100 ms-long windows.

The event related potentials of the four groups at the three parieto-occipital ROIs are shown below in Figure 2.

#### P1

**FIGURE 3:**
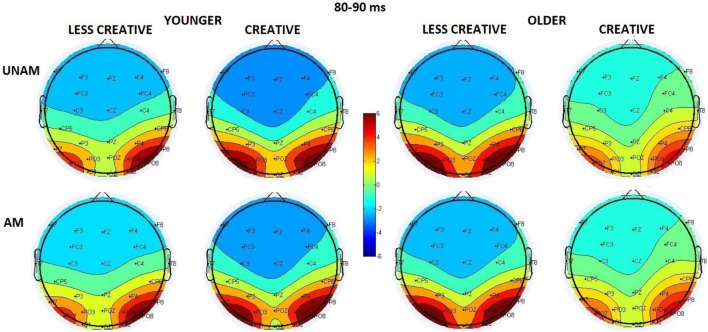
Scalp distribution of the first positive peak in the 80–90 ms range in the four groups (younger – less creative, younger – creative, older – less creative, older – creative) for unambiguous (UNAM, top row) and ambiguous (AM, bottom row) stimuli.

We analyzed the P1 component at the three parieto-occipital ROIs. Older adults had shorter latency than younger adults (*Age* main effect: *F*(1,44) = 4.31, *p* = 0.044, η_p_^2^ = 0.09); but according to the *post hoc* test of the *ROI x Age* interaction (*F*(2,88) = 5.03, *p* = 0.009, η_p_^2^ = 0.10) this effect was significant only at the middle parieto-occipital ROI (*p* = 0.022), where the P1 latency was the longest (*ROI* main affect for latency: *F*(2,88) = 9.84, *p* < 0.001, η_p_^2^ = 0.18). However, as Figure 3 shows, the positivity between 80–90 ms was seen at the left and right ROIs, and not in the middle, where the amplitude was smallest (*ROI* main affect for amplitude: *F*(2,88) = 34.57, *p* < 0.001, η_p_^2^ = 0.44). Nevertheless, the *post hoc* test of the *Stimulus x ROI* interaction (*F*(2,88) = 3.44, *p* = 0.036, η_p_^2^ = 0.07) showed a significant difference between amplitudes of unambiguous and ambiguous trials, and also only at the middle ROI (*p* = 0.045).

**FIGURE 4:**
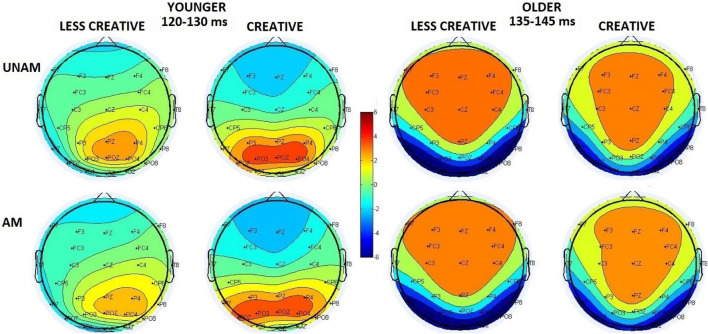
Scalp distribution of the 120–130 ms range in the younger groups (less creative and creative) and of the 135–145 ms range in the older groups (less creative and creative) for unambiguous (UNAM, top row) and ambiguous (AM, bottom row) stimuli.

Instead of analyzing the peak latency and amplitude of the N1 component, we measured P1-N1 peak to peak and N1- 200–300 ms positivity peak to peak amplitude due to the superimposed positivity on the N1 component. The effect of this superimposition could be seen on Figures 2, 4: the negative deflection after the P1 did not reach negative values in the younger groups. It should be noted, when at least two ERP components are suspected to be superimposed, the potential at the peak latency of a wave may not be regarded as the potential of a particular ERP component, so the scalp distributions on Figure 4 are merely potential maps of the related ERPs in the indicated latency ranges (and this can also be assumed for Figure 3).

#### P1–N1 Peak to Peak

The amplitude between the peaks was larger in the older group than in the younger group (*Age* main effect: *F*(1,44) = 12.58, *p* = 0.001, η_p_^2^ = 0.22). The *post hoc* test of the *ROI x Age* interaction (*F*(2,88) = 9.46, *p* < 0.001, η_p_^2^ = 0.18) showed that the amplitude deflection in the older group was larger compared to the younger group at the left (*p* < 0.001) and right (*p* = 0.006) parieto-occipital ROIs. In the younger group the amplitude between the peaks was lager at the right than at the middle parieto-occipital ROI (*p* = 0.038). In the older group the amplitude between the peaks was lager at the left (*p* < 0.001) and right (*p* < 0.001) ROIs than at the middle ROI. *Creativity* had no significant influence to this amplitude difference.

#### N1– 200-300 ms Positivity Peak to Peak

The amplitude between the peaks was larger at the left- (*p* < 0.001) and right (*p* < 0.001) ROIs than at the middle ROI (*ROI* main effect: *F*(2,88) = 44.70, *p* < 0.001, η_p_^2^ = 0.50). The *post hoc* test of the *ROI x Age* interaction (*F*(2,88) = 7.35, *p* = 0.001, η_p_^2^ = 0.14) showed that this was the case in the older group, but in the younger group the amplitude deflection was larger at the right parieto-occipital ROI, than at the left (*p* = 0.011) and middle (*p* < 0.001) ROIs. The *post hoc* test of the *ROI x Creativity* interaction (*F*(2,88) = 4.28, *p* = 0.027, η_p_^2^ = 0.09) did not show significant differences between the less creative and creative groups. According to the *post hoc* test of the *Stimulus x ROI* interaction (*F*(2,88) = 12.47, *p* < 0.001, η_p_^2^ = 0.22) the amplitude for unambiguous trials was larger than for ambiguous trials at the right parieto-occipital ROI (*p* < 0.001), but not differed at the left and middle ROIs.

After 200 ms we analyzed mean amplitudes within 200–300, 300–400, 400–500 and 500–600 ms ranges at the parieto-occipital ROIs, where we found a long-lasting positivity.

#### 200-300 ms Range

**FIGURE 5:**
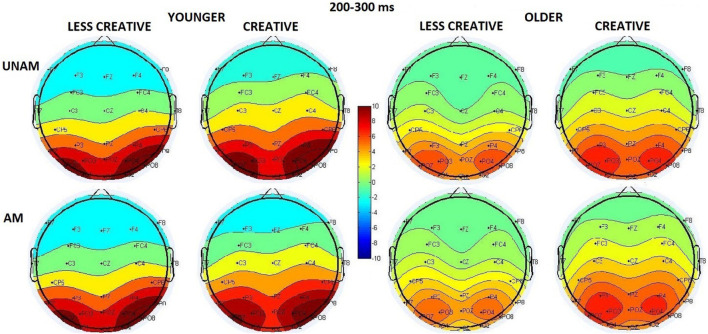
Scalp distribution for the 200–300 ms range in the four groups (younger – less creative, younger – creative, older – less creative, older – creative) for unambiguous (UNAM, top row) and ambiguous (AM, bottom row) stimuli.

The amplitude was larger in the younger compared to the older group (*Age* main effect: *F*(1,44) = 15.09, *p* < 0.001, η_p_^2^ = 0.26), and the amplitude increased from the middle to the left and the right ROI order (*ROI* main effect: *F*(2,88) = 28.61, *p* < 0.001, η_p_^2^ = 0.39 – see scalp distribution in Figure 5), but according to the *post hoc* test of the *ROI x Age* interaction (*F*(2,88) = 12.12, *p* < 0.001, η_p_^2^ = 0.22) this was significant only in the younger group, while no amplitude differences were seen between the ROIs in the older group. The *post hoc* test of the *Stimulus x ROI* interaction (*F*(2,88) = 6.61, *p* = 0.002, η_p_^2^ = 0.13) showed that the amplitude was larger for unambiguous than ambiguous trials at the right parieto-occipital ROI (*p* < 0.001).

#### 300–400 ms Range

**FIGURE 6:**
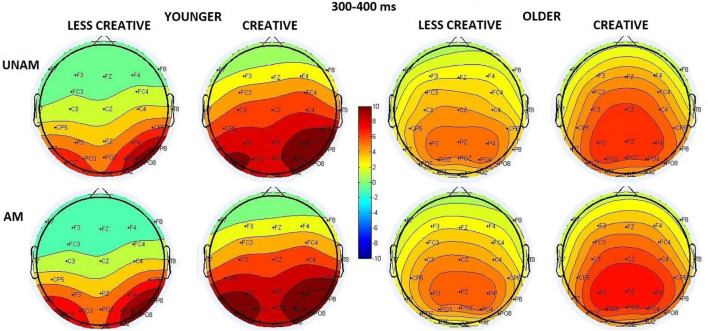
Scalp distribution for the 300–400 ms range in the four groups (younger – less creative, younger – creative, older – less creative, older – creative) for unambiguous (UNAM, top row) and ambiguous (AM, bottom row) stimuli.

The scalp distribution in this time window is shown in Figure 6: the amplitude increased in the younger group from the middle to the left and the right ROI in a corresponding magnitude; while no amplitude differences were seen between the ROIs in the older group (*ROI x Age* interaction: *F*(2,88) = 19.94, *p* < 0.001, η_p_^2^ = 0.31). The amplitude was larger in the younger compared to the older group (*Age* main effect: *F*(1,44) = 11.59, *p* = 0.001, η_p_^2^ = 0.21), and for ambiguous compared to unambiguous trials (*Stimulus* main effect: *F*(1,44) = 5.83, *p* = 0.020, η_p_^2^ = 0.12). The latter effect was significant only in the younger (*p* = 0.008), but not in the older group (*p* = 0.999) according to the *post hoc* test of the *Stimulus x Age* interaction (*F*(1,44) = 5.63, *p* = 0,022, η_p_^2^ = 0.11). The *Stimulus x ROI* interaction (*F*(2,88) = 5.88, *p* = 0.004, η_p_^2^ = 0.12) showed that the amplitude was higher for ambiguous when compared with unambiguous portraits on the left and the right sites, but not in the middle. The *Stimulus x ROI x Age* interaction was also significant (*F*(2,88) = 9.83, *p* < 0.001, η_p_^2^ = 0.18) showing that this effect was apparent in the younger but not in the older groups, where the two types of stimuli did not differ at either ROIs. We found a tendency for *Creativity* main effect (*F*(1,44) = 3.80, *p* = 0.058, η_p_^2^ = 0.08), which showed that the amplitude was larger in the creative groups compared to the less creative groups. Although, the *Age x Creativity* interaction was not significant (*F*(1,44) = 0.30, *p* = 0.589, η_p_^2^ = 0.01), the *post hoc* test did show a significant difference between the creative younger and older groups (*p* = 0.037) but not between the less creative age groups (*p* = 0.195).

#### 400–500 ms Range

**FIGURE 7:**
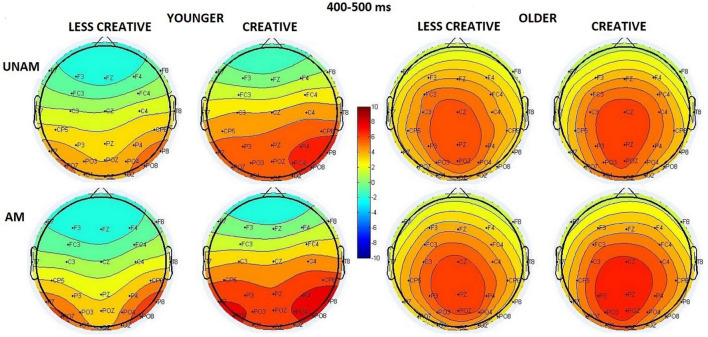
Scalp distribution for the 400–500 ms range in the four groups (younger – less creative, younger – creative, older – less creative, older – creative) for unambiguous (UNAM, top row) and ambiguous (AM, bottom row) stimuli.

We did not find an *Age* main effect in this time window, but the *Age x ROI* interaction (*F*(2,88) = 24.54, *p* < 0.001, η_p_^2^ = 0.36) showed that the scalp distribution differed in the two age groups (Figure 7): in young adults the amplitude had a right-sided maximum (*p*(right vs. middle ROI) < 0.001), while in older adults a middle-sided maximum (*p*(middle vs left and right ROIs) < 0.001) was observed. The amplitude was larger for ambiguous than unambiguous trials (*Stimulus* main effect: *F*(1,44) = 20.45, *p* < 0.001, η_p_^2^ = 0.32). The *post hoc* test of the *Stimulus x Age* interaction (*F*(1,44) = 1.16, *p* = 0.288, η_p_^2^ = 0.03) indicated that the amplitude was larger for ambiguous than unambiguous portraits in younger adults (*p* = 0.002), but only a tendency was seen in the elderly (*p* = 0.085). The *Stimulus x ROI x Age* interaction (*F*(2,88) = 8.73, *p* < 0.001, η_p_^2^ = 0.17) indicated that in younger adults the amplitude was largest at the right ROI when compared with the left and middle ROIs for the unambiguous portraits (*p*(right vs. left and middle ROIs) < 0.001), while it increased in the middle, left, right ROI order for the ambiguous trials (*p*(middle vs. left and right ROIs) < 0.001; *p*(left vs. right ROI) < 0.001). In older adults the amplitude showed its maximum at the middle site for both types of the stimuli (*p* < 0.001 in all cases). We found a tendency for *Creativity* main effect (*F*(1,44) = 2.92, *p* = 0.095, η_p_^2^ = 0.06), which showed that the amplitude was larger in the creative groups compared to the less creative groups. Albeit the *Stimulus x Creativity* interaction was not significant (*F*(1,44) = 2.83, *p* = 0.099, η_p_^2^ = 0.06), the *post hoc* test showed significant difference between unambiguous and ambiguous trials in the creative groups (*p* = 0.001), but not in the less creative groups (*p* = 0.201). The *Stimulus x Creativity x ROI* interaction (*F*(2,88) = 4.06, *p* = 0.021, η_p_^2^ = 0.08) indicated that in the less creative group the amplitude was larger at the middle when compared with the left and right ROI for the unambiguous portraits; and larger at the middle and right ROIs when compared with the left site for the ambiguous trials. In the creative group the amplitude was larger at middle and right ROIs compared to the left ROI for the unambiguous pictures, while no differences were found in the distribution for the ambiguous portraits (*post hoc p* < 0.001 in all cases).

#### 500–600 ms Range

**FIGURE 8:**
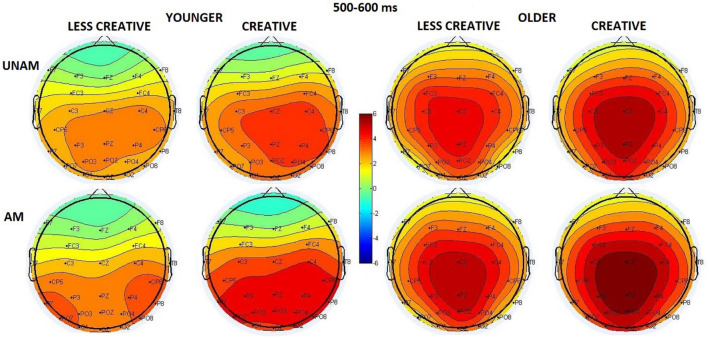
Scalp distribution for the 500–600 ms range in the four groups (younger – less creative, younger – creative, older – less creative, older – creative) for unambiguous (top row) and ambiguous (bottom row) stimuli.

The amplitude was larger for ambiguous than unambiguous trials (*Stimulus* main effect: *F*(1,44) = 25.92, *p* < 0.001, η_p_^2^ = 0.37). The amplitude was the largest at the middle ROI (*ROI* main effect: *F*(2,88) = 18.45, *p* < 0.001, η_p_^2^ = 0.30), but the *ROI x Age* interaction (*F*(2,88) = 17.22, *p* < 0.001, η_p_^2^ = 0.28) showed that it was apparent in the older group (*p*(middle vs left and right) < 0.001), while the three regions did not differ in the younger group (Figure 8). The *Stimulus x ROI x Age* interaction (*F*(2,88) = 11.70, *p* < 0.001, η_p_^2^ = 0.21) indicated that in young adults the amplitude was larger at the middle and right compared to the left ROI for unambiguous pictures, and at the right compared to the middle ROI for the ambiguous portraits. In older adults the maximum was seen at the middle sites for both types of stimuli (*post hoc p* < 0.001 in all cases). The *Stimulus x ROI x Creativity* (*F*(2,88) = 3.48, *p* = 0.035, η_p_^2^ = 0.07) interaction showed a small difference in the scalp distribution: in the creative group the order of the amplitude increased in the left, then the right, and then the middle ROIs for unambiguous pictures; while in the remaining three conditions it was largest at the middle site, and the left and right ROIs did not differ (*post hoc p* < 0.001 in all cases).

### Decoding Analyses

**FIGURE 9:**
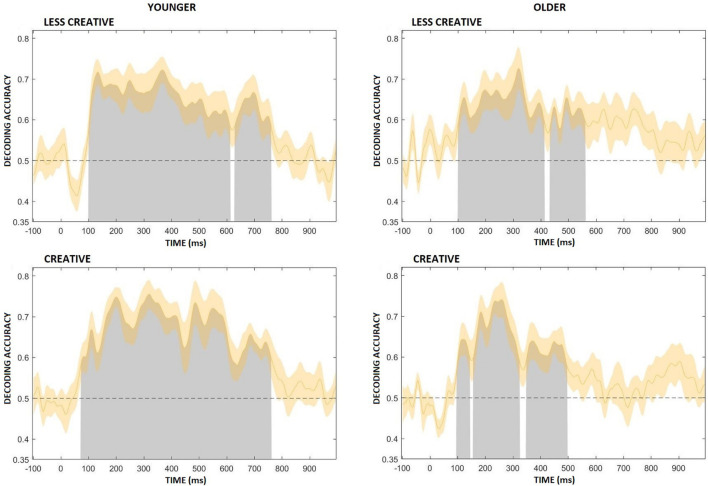
Average decoding accuracy (averaged across participants) for the four groups (younger – less creative, younger – creative, older – less creative, older – creative). Chance level performance (0.5) is indicated by the black horizontal dashed lines. Gray areas indicate clusters of time points in which the decoding was significantly greater than chance. The orange shading indicates ± 1 SEM.

Figure 9 shows average decoding accuracy in the four groups. Decoding was significantly above chance during most of the post-stimulus period in the younger groups. In the younger less creative group decoding accuracy began to rise above chance at ∼100 ms after the onset of the stimulus, and immediately after ∼100 ms it had a short peak, then a slight decrease followed by a longer peak at between ∼300–400 ms. It remained high and significant until ∼760 ms, and then had a third peak at around 700 ms, which also included a short (∼616–632 ms) non-significant period. In the younger more creative group decoding accuracy started to rise above chance at ∼70 ms after the onset of the stimulus, and it lasted, significantly, until ∼765 ms, having peaked multiple times. Decoding was significantly above chance during shorter time intervals in the older groups compared to the younger groups, and short non-significant sections were also included. In the older less creative group decoding accuracy began to rise above chance at ∼100 ms after the onset of the stimulus, and then peaked at around ∼325 ms, and remained significant until ∼565 ms, which included a short (∼415–435 ms) non-significant period. In the older creative group decoding accuracy started to rise above chance at ∼100 ms after the onset of the stimulus, and peaked between ∼200–300 ms, then lasted, significantly, until ∼500 ms; but also included two short (∼150–160 and ∼325–350 ms) non-significant periods.

**FIGURE 10:**
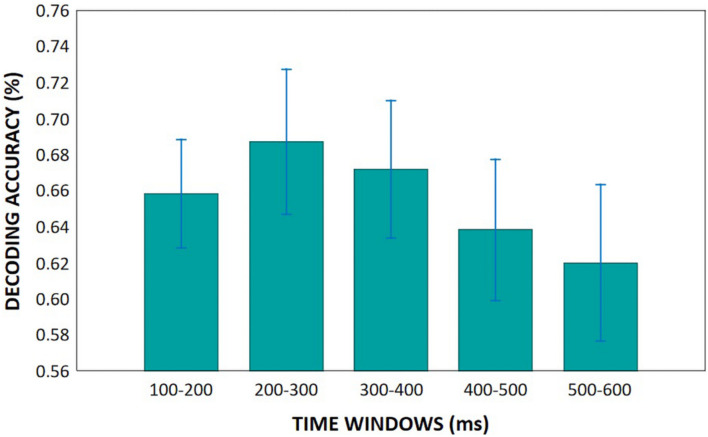
Mean decoding accuracies in the five analyzed time windows. Vertical bars denote 0.95 confidence intervals.

Within the following five periods (100–200, 200–300, 300–400, 400–500, 500–600 ms) almost every groups had statistically significant results, which were greater than chance decoding accuracy, so we aggregated decoding accuracy across time points to be able to compare our groups decoding accuracy. This means, in each time period we averaged the decoding accuracy across the given time points for each participant. Then we performed repeated measures of ANOVAs. We found no differences related to creativity and only tendency level difference between the younger and older groups (*Age* main effect: *F*(1,44) = 3.11, *p* = 0.085, η_p_^2^ = 0.07), which showed that younger adults had greater decoding accuracy compared to older adults. According to the *Time* main effect (*F*(4,176) = 3.62, *p* = 0.007, η_p_^2^ = 0.08), decoding accuracy was higher between 200–300 ms after stimulus onset than within the 500–600 ms range (Figure 10).

## Discussion

The aim of our study was to examine if creativity influences the perception of visual stimuli, or any stages of visual processing; as well as to study, whether healthy aging has an effect on these processes. We hypothesized that decreased cognitive control in older adults would affect their visual processing: as a result of their less effective inhibition they were likely to perceive more details from ambiguous images. To test our hypotheses, we investigated event related potentials and applied ERP decoding analyses in four groups: younger less creative, younger creative, older less creative and older creative adults.

In our experiment we used unambiguous and ambiguous portrait paintings as standard stimuli in an active oddball paradigm. Ambiguous stimuli have an interesting property: although the visual information does not change, the visual perception changes spontaneously from one percept to the other (Kornmeier and Bach, 2012). This is an outstanding tool for revealing the differences between the observers’ brain processes, among them inhibitory functions.

We separated early bottom-up and later top-down processes by analyzing ERP components. The first sensory component we measured, the P1, emerged above the lateral parieto-occipital areas between 30–130 ms after stimulus presentation in both groups, as a correlate of the primary visual response. At those locations where the component showed its amplitude maximum, there was no difference between age groups, creativity groups or the two types of stimuli. This suggests that the physical properties of the two portrait categories did not differ significantly for the visual system; and also, neither aging nor creativity influenced the participants’ early perceptual processing.

Instead of analyzing the N1 component, we measured peak to peak amplitudes before and after it, since the extent of temporal overlapping of the N1 and the subsequent components differed between the age groups. Creativity had no effect on neither P1-N1 peak-to-peak nor N1- 200–300 ms positivity peak to peak amplitude deflection. These, with the above results about the P1 show that creativity did not influence the early stage of visual processing. By analyzing the positivity in 100 ms-long time windows after 200 ms, our goal was to characterize the top-down processes in fine resolution. An aging effect was found between 200–400 ms, being that the amplitude was lower in the elderly. This result, especially in the time range of the P3 component, with slower reaction times, have been frequently observed (pl. Polich, 1997; Gaál et al., 2007), which may be due to the general age-related cognitive decline, caused by impaired inhibitory processes (Hasher and Zacks, 1988) and their slower processing speed (Salthouse, 1996).

The question that concerned us most was whether creativity influenced the processing of visual stimuli. In answer to this we found that the amplitude was larger between 300 and 500 ms in the creative groups when compared with the less creative groups. This time period overlaps with the time window of the P3b component, which suggests a categorization difference in top-down processing between creative and less creative people, the former being more effective in these processes. Age also influenced this effect: between 300-400 ms younger adults had a larger amplitude when compared with older adults in the creative group, but no such difference was found between the less creative groups. These results suggest that younger adults are the standout group in this regard.

In addition to looking at the effects of aging and creativity in itself, we were interested to know whether processing of the two types of stimuli differ, and whether creativity or age influence this processing. In examining the two types of stimuli, we had assumed that there was a special relationship between the whole and its parts for the ambiguous stimuli as they were more interesting, and complex.

Despite both unambiguous and ambiguous images being irrelevant to the task (as participants did not have to respond to these stimuli), they still elicited large ERP amplitudes. This is not typical with standard stimuli in traditional oddball paradigms, however, the stimuli we used are uncommon to these by being not just simple geometric shapes, such as circles or rectangles, but visually interesting and therefore salient images. So this large amplitude observed could be related to the complexity of the paintings (Barkaszi et al., 2013).

Beyond the fact that our standard stimuli elicited large amplitudes, the two types of stimuli were clearly distinguishable. Between 200–300 ms the amplitude was larger for unambiguous than ambiguous portraits (which was also seen for P1 component, but not at that ROIs where it reached its maximum); while from 300 ms to the end of the analyzed period the amplitude was larger for ambiguous than unambiguous paintings. The latter difference in the traditional analysis was observable in the younger, but not older groups between 300–500 ms. Creativity of the participants had no observed influence on this result.

A possible explanation for the larger amplitude elicited by ambiguous portraits observed in younger groups is based on Navon’s global precedence hypothesis (Navon, 1977), which claims that “the whole” takes priority over the individual parts during visual processing. Roux and Ceccaldi (2001) examined age-related changes in the global precedence effect in a selective attention task (identifying letters globally or locally); and although the effect was detectable in both younger and older adults, a greater global interference effect was observed in the elderly when identifying local letters. On the one hand, this could be caused by the age-related decline of inhibitory control, and on the other, it is possible that global-local processing may take place differently in the elderly. This is supported by Gottlob and Madden (1999), who claimed that older people can only process in a sequential way, first on a global, then to a local level; while younger people can process both parallely. These assumptions are fairly consistent with the results of studies in which during passive viewing of bistable images (Rubin’s vase, Necker cube) the duration of dominant perceptions was longer in older compared to younger adults as they had less perception changes between the two alternatives (Aydin et al., 2013; Díaz-Santos et al., 2017; Kondo and Kochiyama, 2018). It is of note that in these studies the images were simpler and were presented for a much longer time (30 s, 60 s, and 5 min) than in our experiment (300 ms), albeit with fewer repetitions.

Taking the above into consideration, the ambiguous images we used can be interpreted either globally or locally depending upon the ability to shift focus from one to the other. An important factor in seeing though this ambiguity is having cognitive control and the flexibility of attention. So, contrary to our hypothesis that the decline in inhibitory control may lead to increased processing of irrelevant stimuli (Lavie, 2005; Biss et al., 2013), especially the processing of more details with the ambiguous portraits, our results suggested that there was no attentional shift in the elderly from global to local images. We believe this was probably due to another mechanism: as in bistable perception only one percept is visible at one time with the other being inhibited, so older adults may have not suppressed their first interpretation of the paintings, and did not switch to the other (Wiseman et al., 2011; Alais, 2012). Also, the 300 ms long presentation of the images was insufficient time for the elderly to make this switch.

In contrast with traditional ERP analysis which only measures the components at their maximum amplitude, our results using three ROIs showed significant differences at other electrode locations, where the component did not reach its maximum (e.g., P1 and N1 amplitudes showed differences between unambiguous and ambiguous portraits where the scalp distribution showed low amplitudes). Furthermore, as decoding is a more sensitive method, because it can utilize information that would not be detectable when comparing ERPs in a univariate (traditional) analysis (Grootswagers et al., 2017; Hebart and Baker, 2018), we additionally performed decoding analysis in an explorative manner. Although this method was originally developed for comparing two conditions, decoding can also be used for group comparisons (the idea based on Bae et al., 2020).

In previous studies decoding methods have become increasingly popular for time series of neuroimaging data using temporal characterization of visual stimuli and object category processing (e.g., Carlson et al., 2011, 2013; Cauchoix et al., 2014; Carlson and Wardle, 2015). While some studies applied MVPA to assess the temporal dynamics of face identity processing (e.g., Nemrodov et al., 2016, 2018; Vida et al., 2017), other, more recent studies have shown that representations of visual stimuli can be decoded during a working memory task, either by examining the spatial pattern of alpha-band EEG oscillations (e.g., Foster et al., 2016; Bae and Luck, 2018) or by using the scalp distribution of event-related potentials (ERPs; e.g., LaRocque et al., 2013; Wolff et al., 2017; Bae and Luck, 2018). These studies have shown that not only stimulus categories but also feature values along continuous dimensions (e.g., orientation in Bae and Luck, 2018; motion direction in Bae and Luck, 2019b).

The sensitivity of the decoding analysis was supported by our results as it showed that the neural signal already contained information about the stimulus type from around 100 ms after the stimulus onset. Furthermore, our results showed that the stimulus type representations could be decoded from scalp EEG activity not just in the younger age groups, but also in the elderly, which would suggest that there was some level of discrimination between the two stimuli type within the 300–500 ms interval, but was not evident in the ERP results. In fact, an above-chance decoding accuracy meant that there was some activity in the neural signal that correlated with the stimulus category. We cannot be certain, however, from our results, whether the classifier distinguished between the two stimuli types were based on ambiguity or else, on a confounding factor that covaries with stimulus/trial type (Grootswagers et al., 2017; Bae and Luck, 2018).

Another unexpected result was that while ERP analysis showed that the amplitudes elicited by the two stimuli type differed between 500 and 600 ms in both the younger and in the older groups, decoding accuracy in the elderly was significant only in the less creative group between 500–565 ms; whereas in the creative group it was not significant at all. This might question our ERP result which we obtained within the 500–600 ms range in the elderly groups. However, it is of note that data from only 13 individual electrodes were analyzed in the ERP analysis, while all of the 27 electrodes and their relationship (i.e., activity pattern across electrodes) were taken into account during ERP decoding, so within this time window, more electrodes with more dimensions could have reduced the decoding accuracy (Grootswagers et al., 2017). With the positive component after 200 ms, we found the waves between younger and older groups differed - a shorter peak and an earlier onset decline in the elderly, while a more prolonged positivity appeared in the younger groups. Here, the decoding accuracy showed a similar pattern as the representation of stimuli lasted longer in younger adults, which might suggest that they were more interested in the stimuli.

In summary, we could not prove that creativity influences the early stage of perception. A limitation of the current study is the relatively small sample size; therefore, the discussed results should be treated with caution. Furthermore, in a cross-sectional study we cannot estimate the causal relationships; a longitudinal study would be necessary to track how creativity changes as a function of age-related decline in inhibitory processes, or whether other latent variables influence group differences. As a first step, using a domain-specific approach, we only narrowed our study to visual creativity. As creativity is a complex construct, in a further study we would divide the groups along cognitive styles; as possibly the adaptive and innovative component of creativity may influence early visual processing differently. Nevertheless, creativity had an effect on stimulus processing within the later processing phase (300-500 ms range) with indexing differences in top-down control, having more flexible cognitive control in the younger creative group. However, we found the older adults had deteriorated inhibitory processes, which not resulted in them having a wider attentional focus and being able to process more details in the ambiguous portrait paintings. Nonetheless, with the elderly having an inability to suppress the global percept, and to change to the processing of details, they demonstrated that the decreased inhibitory control was not an advantage for them.

## Data Availability Statement

The original contributions presented in the study are included in the article/supplementary material, further inquiries can be directed to the corresponding author.

## Ethics Statement

The studies involving human participants were reviewed and approved by Joint Psychological Research Ethics Committee (EPKEB). The participants provided their written informed consent to participate in this study.

## Author Contributions

PC, ZG, and IC designed the study. PC, BN, and ZG collected data. PC, BN, and ZG analyzed data. PC and ZG wrote the manuscript. All authors contributed to the article and approved the submitted version.

## Conflict of Interest

The authors declare that the research was conducted in the absence of any commercial or financial relationships that could be construed as a potential conflict of interest.

## Publisher’s Note

All claims expressed in this article are solely those of the authors and do not necessarily represent those of their affiliated organizations, or those of the publisher, the editors and the reviewers. Any product that may be evaluated in this article, or claim that may be made by its manufacturer, is not guaranteed or endorsed by the publisher.

## Footnotes

1 Note that early and late stages of the ERPs are not identical of the initial and later stages of the creative process. While in the former case also the late stage is within 1000 ms, in the latter case it can last from minutes to months.

2 Nagy, B., Czigler, I., File, D., Csizmadia, P., Fáy, N., and Gaál, Zs. A (under review). Do Age- Related Changes In Cognitive Control Affect Creative Performance In Older Compared To Younger Adults?-An Erp Study Of Task-Switching.

3 Unambiguous portraits were: Anonymous: William Shakespeare, van Gogh: Self-portrait, Michelangelo: Self-portrait, Bondar: Leo Tolstoy. Ambiguous portraits were: Oleg Shuplyak: William Shakespeare, Vincent van Gogh, Prophet Michelangelo, Anna Karenina (Leo Tolstoy) - with the permission of the artist.

4
https://osf.io/29wre/

## References

[B1] Alais D. (2012). Binocular rivalry: competition and inhibition in visual perception. *Wiley Interdiscip. Rev. Cogn. Sci.* 3 87–103. 10.1002/wcs.151 26302474

[B2] Aydin S. Strang N. C. Manahilov V. (2013). Age-Related deficits in attentional control of perceptual rivalry. *Vision Res.* 77 32–40. 23206550 10.1016/j.visres.2012.11.010

[B3] Bae G. Y. Luck S. J. (2018). Dissociable decoding of spatial attention and working memory from EEG oscillations and sustained potentials. *J. Neurosci.* 38 409–422. 10.1523/JNEUROSCI.2860-17.2017 29167407 PMC5761617

[B4] Bae G. Y. Luck S. J. (2019a). Appropriate correction for multiple comparisons in decoding of erp data: a re-analysis of bae & luck (2018). *bioRxiv* [Preprint]. 10.1101/672741

[B5] Bae G. Y. Luck S. J. (2019b). Decoding motion direction using the topography of sustained ERPs and alpha oscillations. *Neuroimage* 184 242–255. 10.1016/j.neuroimage.2018.09.029 30223063 PMC6230491

[B6] Bae G. Y. Luck S. J. (2019c). Reactivation of previous experiences in a working memory task. *Psychol. Sci.* 30 587–595. 10.1177/0956797619830398 30817224 PMC6472175

[B7] Bae G. Y. Leonard C. J. Hahn B. Gold J. M. Luck S. J. (2020). Assessing the information content of ERP signals in schizophrenia using multivariate decoding methods. *Neuroimage. Clin.* 25:102179. 10.1016/j.nicl.2020.102179 31954988 PMC6965722

[B8] Baer J. (2015). The importance of domain-specific expertise in creativity. *Roeper Rev.* 37 165–178. 10.1080/02783193.2015.1047480

[B9] Barkaszi I. Czigler I. Balázs L. (2013). Stimulus complexity effects on the event-related potentials to task-irrelevant stimuli. *Biol. Psychol.* 94 82–89. 10.1016/j.biopsycho.2013.05.007 23702457

[B10] Barkóczi I. Klein D. (1968). Gondolatok az alkotóképességről és vizsgálatának problémáiról. *Magyar Pszichológiai Szemle* 25 508–515.

[B11] Barkóczi I. Zétényi T. (1981). *A kreativitás vizsgálata. Pszichológiai tanácsadás a pályaválasztásban. Módszertani füzetek 2.* Budapest: Országos Pedagógiai Intézet.

[B12] Bentin S. Allison T. Puce A. Perez E. McCarthy G. (1996). Electrophysiological studies of face perception in humans. *J. Cogn. Neurosci.* 8 551–565. 10.1162/jocn.1996.8.6.551 20740065 PMC2927138

[B13] Biss R. K. Ngo K. W. Hasher L. Campbell K. L. Rowe G. (2013). Distraction can reduce age-related forgetting. *Psychol. Sci.* 24 448–455. 10.1177/0956797612457386 23426890

[B14] Carlson T. A. Wardle S. G. (2015). Sensible decoding. *Neuroimage* 110 217–218. 10.1016/j.neuroimage.2015.02.009 25680521

[B15] Carlson T. A. Hogendoorn H. Kanai R. Mesik J. Turret J. (2011). High temporal resolution decoding of object position and category. *J. Vis.* 11:9. 10.1167/11.10.9 21920851

[B16] Carlson T. Tovar D. A. Alink A. Kriegeskorte N. (2013). Representational dynamics of object vision: the first 1000 ms. *J. Vis.* 13:1. 10.1167/13.10.1 23908380

[B17] Cauchoix M. Barragan-Jason G. Serre T. Barbeau E. J. (2014). The neural dynamics of face detection in the wild revealed by MVPA. *J. Neurosci.* 34 846–854. 10.1523/JNEUROSCI.3030-13.2014 24431443 PMC6608346

[B18] Chamola V. Vineet A. Nayyar A. Hossain E. (2020). Brain-Computer Interface-Based Humanoid Control: a Review. *Sensors (Basel, Switzerland)* 20:3620. 10.3390/s20133620 32605077 PMC7374399

[B19] Chrysikou E. G. Weber M. J. Thompson-Schill S. L. (2014). A matched filter hypothesis for cognitive control. *Neuropsychologia* 62 341–355. 10.1016/j.neuropsychologia.2013.10.021 24200920 PMC4010565

[B20] Curran E. A. Stokes M. J. (2003). Learning to control brain activity: a review of the production and control of EEG components for driving brain-computer interface (BCI) systems. *Brain Cogn.* 51 326–336. 10.1016/s0278-2626(03)00036-8 12727187

[B21] Czigler I. Cox T. J. Gyimesi K. Horváth J. (2007). Event-related potential study to aversive auditory stimuli. *Neurosci. Lett.* 420 251–256. 10.1016/j.neulet.2007.05.007 17556101

[B22] Delorme A. Makeig S. (2004). EEGLAB: an open source toolbox for analysis of single-trial EEG dynamics including independent component analysis. *J. Neurosci. Methods* 134 9–21. 10.1016/j.jneumeth.2003.10.009 15102499

[B23] Díaz-Santos M. Mauro S. Cao B. Yazdanbakhsh A. Neargarder S. Cronin-Golomb A. (2017). Bistable perception in normal aging: perceptual reversibility and its relation to cognition. *Neuropsychol. Dev. Cogn. B Aging Neuropsychol. Cogn.* 24 115–134. 10.1080/13825585.2016.1173646 27116194 PMC5467698

[B24] Dietrich A. Kanso R. (2010). A review of EEG, ERP, and neuroimaging studies of creativity and insight. *Psychol. Bull.* 136 822–848. 10.1037/a0019749 20804237

[B25] Dietterich T. G. Bakiri G. (1995). Solving multiclass learning problems via error-correcting output codes. *J. Artif. Intell. Res.* 2 263–286. 10.1613/jair.105

[B26] Fáy N. Jeney Á. (2016). “A kreativitás-mérés kiértékelésének megújítása (Renewing the evaluation of creativity measurement),” in *A Magyar Pszichológiai Társaság XXV*. ed. Vargha A. (Budapest: Országos Tudományos Nagygyülése: program és elöadáskivonatok, 58.

[B27] Fáy N. Kovács A. J. Kollár N. Jeney Á (in press). *A Megújított Barkóczi-Klein Kreatív Potenciál-Teszt Kialakítása, Módszertana És Fõbb Eredményei Az Országos Mintán. Alkalmazott Pszichológia.* Alkalmazott Pszichológia.

[B28] Foster J. J. Sutterer D. W. Serences J. T. Vogel E. K. Awh E. (2016). The topography of alpha-band activity tracks the content of spatial working memory. *J. Neurophysiol.* 115 168–177. 10.1152/jn.00860.2015 26467522 PMC4760461

[B29] Gaál Zs. A Csuhaj R. Molnár M. (2007). Age-dependent changes of auditory evoked potentials–effect of task difficulty. *Biol. Psychol.* 76 196–208. 10.1016/j.biopsycho.2007.07.009 17767993

[B30] Gottlob L. R. Madden D. J. (1999). Age differences in the strategic allocation of visual attention. *J. Gerontol. B Psychol. Sci. Soc. Sci.* 54 165–172. 10.1093/geronb/54b.3.p165 10363038

[B31] Grootswagers T. Wardle S. G. Carlson T. A. (2017). Decoding dynamic brain patterns from evoked responses: a tutorial on multivariate pattern analysis applied to time series neuroimaging data. *J. Cogn. Neurosci.* 29 677–697. 10.1162/jocn_a_01068 27779910

[B32] Guilford J. P. (1967). *The Nature of Human Intelligence.* New York, NY: McGraw-Hill.

[B33] Hasher L. Zacks R. T. (1988). “Working memory, comprehension, and aging: A review and a new view,” in *The Psychology Of Learning And Motivation: Advances In Research And Theory*, Vol. 22 ed. Bower G. H. (Cambridge, MA: Academic Press), 193–225. 10.1016/S0079-7421(08)60041-9

[B34] Haynes J. D. (2015). A primer on pattern-based approaches to fMRI: principles. Pitfalls, and perspectives. *Neuron* 87 257–270. 10.1016/j.neuron.2015.05.025 26182413

[B35] Healey M. K. Campbell K. L. Hasher L. (2008). Cognitive aging and increased distractibility: costs and potential benefits. *Prog. Brain Res.* 169 353–363. 10.1016/S0079-6123(07)00022-2 18394486

[B36] Hebart M. N. Baker C. I. (2018). Deconstructing multivariate decoding for the study of brain function. *Neuroimage* 180 (Pt A) 4–18. 10.1016/j.neuroimage.2017.08.005 28782682 PMC5797513

[B37] Hillyard S. A. Vogel E. K. Luck S. J. (1998). Sensory gain control (amplification) as a mechanism of selective attention: electrophysiological and neuroimaging evidence. *Philos. Trans. R. Soc. Lond. B Biol. Sci.* 353 1257–1270. 10.1098/rstb.1998.0281 9770220 PMC1692341

[B38] Hommel B. (2015). Between persistence and flexibility: the Yin and Yang of action control. *Adv. Motiv. Sci.* 2 33–67. 10.1016/bs.adms.2015.04.003

[B39] Hopf J. M. Vogel E. Woodman G. Heinze H. J. Luck S. J. (2002). Localizing visual discrimination processes in time and space. *J. Neurophysiol.* 88 2088–2095. 10.1152/jn.2002.88.4.2088 12364530

[B40] Kim S. Rasher L. Zacks R. T. (2007). Aging and a benefit of distractibility. *Psychon. Bull. Rev.* 14 301–305. 10.3758/bf03194068 17694917 PMC2121579

[B41] Kondo H. M. Kochiyama T. (2018). Normal aging slows spontaneous switching in auditory and visual bistability. *Neuroscience* 389 152–160. 10.1016/j.neuroscience.2017.04.040 28479403

[B42] Kornmeier J. Bach M. (2012). Ambiguous figures - what happens in the brain when perception changes but not the stimulus. *Front. Hum. Neurosci.* 6:51. 10.3389/fnhum.2012.00051 22461773 PMC3309967

[B43] LaRocque J. J. Lewis-Peacock J. A. Drysdale A. T. Oberauer K. Postle B. R. (2013). Decoding attended information in short-term memory: an EEG study. *J. Cogn. Neurosci.* 25 127–142. 10.1162/jocn_a_00305 23198894 PMC3775605

[B44] Lavie N. (2005). Distracted and confused?: selective attention under load. *Trends Cogn. Sci.* 9 75–82. 10.1016/j.tics.2004.12.004 15668100

[B45] Long G. M. Toppino T. C. (2004). Enduring interest in perceptual ambiguity: alternating views of reversible figures. *Psychol. Bull.* 130:748768. 10.1037/0033-2909.130.5.748 15367079

[B46] Luck S. J. (2014). *An Introduction to the Event-Related Potential Technique.* Cambridge, MA: MIT Press.

[B47] Luck S. J. Woodman G. F. Vogel E. K. (2000). Event-related potential studies of attention. *Trends Cogn. Sci.* 4 432–440. 10.1016/s1364-6613(00)01545-x 11058821

[B48] Mangun G. R. Hillyard S. A. (1990). Allocation of visual attention to spatial locations: tradeoff functions for event-related brain potentials and detection performance. *Percept. Psychophys.* 47 532–550. 10.3758/bf03203106 2367174

[B49] Müller K. R. Tangermann M. Dornhege G. Krauledat M. Curio G. Blankertz B. (2008). Machine learning for real-time single-trial EEG-analysis: from brain-computer interfacing to mental state monitoring. *J. Neurosci. Methods* 167 82–90. 10.1016/j.jneumeth.2007.09.022 18031824

[B50] Navon D. (1977). Forest before trees: the precedence of global features in visual perception. *Cogn. Psychol.* 9 353–383. 10.1016/0010-0285(77)90012-3

[B51] Nęcka E. (2011). “Perception and creativity,” in *Encyclopedia of Creativity*, 2nd Edn, eds Runco M. A. Pritzker S. R. (Cambridge: Elsvier), 216–219. 10.1016/j.tsc.2018.11.003

[B52] Nemrodov D. Niemeier M. Mok J. Nestor A. (2016). The time course of individual face recognition: a pattern analysis of ERP signals. *Neuroimage* 132 469–476. 10.1016/j.neuroimage.2016.03.006 26973169

[B53] Nemrodov D. Niemeier M. Patel A. Nestor A. (2018). The neural dynamics of facial identity processing: insights from EEG-based pattern analysis and image reconstruction. *eNeuro* 5:ENEURO.0358-17.2018. 10.1523/ENEURO.0358-17.2018 29492452 PMC5829556

[B54] Nijstad B. A. De Dreu C. K. W. Rietzschel E. F. Baas M. (2010). The dual pathway to creativity model: creative ideation as a function of flexibility and persistence. *Eur. Rev. Soc. Psychol.* 21 34–77. 10.1080/10463281003765323

[B55] Norman K. A. Polyn S. M. Detre G. J. Haxby J. V. (2006). Beyond mind-reading: multi-voxel pattern analysis of fMRI data. *Trends Cogn. Sci.* 10 424–430. 10.1016/j.tics.2006.07.005 16899397

[B56] Paz-Caballero M. D. García-Austt E. (1992). ERP components related to stimulus selection processes. *Electroencephalogr. Clin. Neurophysiol.* 82 369–376. 10.1016/0013-4694(92)90006-4 1374705

[B57] Polich J. (1997). EEG and ERP assessment of normal aging. *Electroencephalogr. Clin. Neurophysiol.* 104 244–256. 10.1016/s0168-5597(97)96139-6 9186239

[B58] Polich J. (2007). Updating P300: an integrative theory of P3a and P3b. *Clin. Neurophysiol.* 118 2128–2148. 10.1016/j.clinph.2007.04.019 17573239 PMC2715154

[B59] Radel R. Davranche K. Fournier M. Dietrich A. (2015). The role of (dis)inhibition in creativity: decreased inhibition improves idea generation. *Cognition* 134 110–120. 10.1016/j.cognition.2014.09.001 25460384

[B60] Roux F. Ceccaldi M. (2001). Does aging affect the allocation of visual attention in global and local information processing? *Brain Cogn.* 46 383–396. 10.1006/brcg.2001.1296 11487288

[B61] Rózsa S. Kő N. Kuncz E. Mészáros A. Mlinkó R. (2010). *WAIS-IV. Wechsler Adult Intelligence Scale - Fourth Edition. Tesztfelvételi és pontozási kézikönyv. Magyar adaptáció.* Budapest: OS-Hungary Tesztfejlesztõ Kft. Available online at: http://www.oshungary.hu/hu/tesztkatalogus-oshungary/wais-iv/

[B62] Runco M. A. Jaeger G. J. (2012). The standard definition of creativity. *Creat. Res. J.* 24 92–96. 10.1080/10400419.2012.650092

[B63] Rutter B. Kröger S. Hill H. Windmann S. Hermann C. Abraham A. (2012). Can clouds dance? Part 2: an ERP investigation of passive conceptual expansion. *Brain Cogn.* 80 301–310. 10.1016/j.bandc.2012.08.003 23137771

[B64] Salthouse T. A. (1996). The processing-speed theory of adult age differences in cognition. *Psychol. Rev.* 103 403–428. 10.1037/0033-295X.103.3.403 8759042

[B65] Stein M. I. (1953). Creativity and culture. *J. Psychol.* 36 311–322. 10.1080/00223980.1953.9712897

[B66] Stevens C. E. Jr. Zabelina D. L. (2019). Creativity comes in waves: an EEG-focused exploration of the creative brain. *Curr. Opin. Behav. Sci.* 27 154–162. 10.1016/j.cobeha.2019.02.003

[B67] Tong F. Pratte M. S. (2012). Decoding patterns of human brain activity. *Annu. Rev. Psychol.* 63 483–509. 10.1146/annurev-psych-120710-100412 21943172 PMC7869795

[B68] Vida M. D. Nestor A. Plaut D. C. Behrmann M. (2017). Spatiotemporal dynamics of similarity-based neural representations of facial identity. *Proc. Natl. Acad. Sci. U.S.A.* 114 388–393. 10.1073/pnas.1614763114 28028220 PMC5240702

[B69] Vogel E. K. Luck S. J. (2000). The visual N1 component as an index of a discrimination process. *Psychophysiology* 37 190–203. 10.1016/0013-4694(90)90139-B 10731769

[B70] Wallas G. (1926). *The Art Of Thought.* London: J. Cape Ltd.

[B71] Wiseman R. Watt C. Gilhooly K. Georgiou G. (2011). Creativity and ease of ambiguous figural reversal. *Br. J. Psychol.* 102 615–622. 21752010 10.1111/j.2044-8295.2011.02031.x

[B72] Wolff M. J. Jochim J. Akyürek E. G. Stokes M. G. (2017). Dynamic hidden states underlying working-memory-guided behavior. *Nat. Neurosci.* 20 864–871. 10.1038/nn.4546 28414333 PMC5446784

[B73] Zabelina D. L. Ganis G. (2018). Creativity and cognitive control: behavioral and ERP evidence that divergent thinking, but not real-life creative achievement, relates to better cognitive control. *Neuropsychologia* 118(Pt A) 20–28. 10.1016/j.neuropsychologia.2018.02.014 29447843

[B74] Zabelina D. L. Robinson M. D. (2010). Creativity as flexible cognitive control. *Psychol. Aesthet. Creat. Arts* 4 136–143. 10.1037/a0017379

[B75] Zhang W. Sjoerds Z. Hommel B. (2020). Metacontrol of human creativity: the neurocognitive mechanisms of convergent and divergent thinking. *Neuroimage* 210:116572. 10.1016/j.neuroimage.2020.116572 31972282

